# Polish translation, cultural adaptation, and validity confirmation of the Scored Patient-Generated Subjective Global Assessment

**DOI:** 10.1007/s00520-024-08808-5

**Published:** 2024-09-04

**Authors:** Katarzyna Zabłocka-Słowińska, Joanna Pieczyńska, Anna Prescha, Maciej Bladowski, Damian Gajecki, Dorota Kamińska, Katarzyna Neubauer, Faith Ottery, Harriët Jager-Wittenaar

**Affiliations:** 1 The Faculty of Finance and Management, WSB Merito University in Wroclaw, Wroclaw, Poland; 2 Department of Health Sciences, University of Opole, Opole, Poland; 3 Department of Dietetics and Bromatology, Wroclaw Medical University, Wroclaw, Poland; 4 Department of Internal and Occupational Diseases, Hypertension and Clinical Oncology, Wroclaw Medical University, Wroclaw, Poland; 5 Department and Clinic of Nephrology and Transplantation Medicine, Wroclaw Medical University, Wroclaw, Poland; 6 Department and Clinic of Gastroenterology and Hepatology, Wroclaw Medical University, Wroclaw, Poland; 7 Ottery & Associates, LLC, Oncology Care Consultants, Chicago, USA; 8 Research Group Healthy Ageing, Allied Health Care and Nursing, Hanze University of Applied Sciences, Groningen, Netherlands; 9 Department of Gastroenterology and Hepatology, Dietetics, Radboud University Medical Center, Nijmegen, Netherlands; 10 Faculty of Physical Education and Physiotherapy, Department Physiotherapy and Human Anatomy, Research Unit Experimental Anatomy, Vrije Universiteit Brussel, Brussels, Belgium

**Keywords:** PG-SGA, Patient-Generated Subjective Global Assessment; Validity; Nutritional assessment; Malnutrition screening

## Abstract

**Purpose:**

The Scored Patient-Generated Subjective Global Assessment (PG-SGA©) is a validated nutritional screening, assessment, triage, and monitoring tool. The aim of this study was to perform translation, cultural adaptation, linguistic, and content validation of the translated and culturally adapted version of the PG-SGA for the Polish setting.

**Methods:**

The study was performed in concordance with the International Society for Pharmacoeconomics and Outcomes Research (ISPOR) Principles. Patients (*n* = 174) and healthcare professionals (HCPs, *n* = 188) participated in the study. Comprehensibility and difficulty were assessed by patients for the PG-SGA Short Form, and by HCPs for the professional component. Content validity was assessed for the full PG-SGA by HCPs only. Evaluations were operationalized by a 4-point scale. Item and scale indices were calculated using the average item ratings divided by the number of respondents. Item indices < 0.78 required further analysis of the item, while scale indices ≥ 0.90 were defined as excellent and 0.80–0.89 as acceptable.

**Results:**

The PG-SGA Short Form was rated as excellent for content validity (Scale-CVI = 0.90) by HCPs and easy to comprehend (Scale-CI = 0.96) and use (Scale-DI = 0.94) by patients. The professional component of the PG-SGA was perceived as acceptable for content validity (Scale-CVI = 0.80), comprehension (Scale-CI = 0.87), and difficulty (Scale-DI = 0.80). The physical exam was rated the least comprehensible and the most difficult, and with the lowest content validity. We found significant differences in scale indices (*p* < 0.05 for all) between HCPs with different professions and between those being familiar with PG-SGA and not.

**Conclusion:**

Translation and cultural adaptation of the PG-SGA for the Polish setting preserved the purpose and conceptual meaning of the original PG-SGA. Validation revealed that the Polish version of PG-SGA is well understood and easy to complete by patients and professionals, and is considered relevant by professionals. However, detailed results indicate the need for appropriate training of the Polish HCPs, especially physicians and nurses, mainly in the worksheets related to the metabolic demand and physical exam.

## Introduction

Both acute and chronic diseases may lead to malnutrition, which has been defined as “a state resulting from lack of intake or uptake of nutrition that leads to altered body composition (decreased fat-free mass) and body cell mass leading to diminished physical and mental function and impaired clinical outcome from disease” [1, 2]. Disease-related malnutrition increases complication rates and mortality, is also associated with longer hospital stays, and causes an increased financial burden on healthcare organizations [1, 3].

Malnutrition is multifactorial and multidimensional, and adequately identifying malnutrition and its key determinants must be regarded as essential [4, 5]. According to the consensus established by the Global Leadership Initiative on Malnutrition (GLIM), which has been formed by representatives of major clinical nutrition societies including the European Society for Clinical Nutrition and Metabolism (ESPEN), the American Society for Clinical Nutrition and Metabolism (ASPEN), Federación Latinoamericana de Terapia Nutricional, Nutrición Clínica y Metabolismo (FELANPE), and the Parenteral and Enteral Nutrition Society of Asia (PENSA), the process of malnutrition diagnosis includes two steps. The first step is malnutrition risk screening and the second step is nutritional assessment for diagnosis and severity grading of malnutrition. The diagnosis of malnutrition must encompass both phenotypic criteria (i.e., weight loss, low body mass index, and reduced muscle mass) and etiologic criteria (i.e., reduced food intake or absorption and disease burden or inflammation), whereby at least one etiologic criterion and one phenotypic criterion are required to diagnose malnutrition [6]. Consistent with these guidelines are the ESPEN recommendations that the patient’s nutritional care should be provided in a systematic way, which involves identifying malnourished patients or patients at risk of malnutrition using validated screening tools, followed by implementation of adequate intervention and monitoring the results of the intervention [7].

In Poland, the concept of the two-step approach of screening and assessment has been implemented, and accordingly, two tools, i.e., Nutritional Risk Score 2002 (NRS 2002) or Subjective Global Assessment (SGA), are obligatorily used in all Polish hospital wards on the patient admission and then every 14 days of the hospitalization. In elderly patients, Mini Nutritional Assessment (MNA) is also used. The other tool recommended by ESPEN, i.e., the Malnutrition Universal Screening Tool (MUST), is available as well [7, 8]. In a recent study aiming to audit the nutritional status of 10,863 hospitalized patients in 25 European countries, including Poland, another simple and frequently used tool—Malnutrition Screening Tool (MST)—was applied. The prevalence rates of malnutrition risk and malnutrition in Polish hospital wards, based on ESPEN diagnostic criteria [7], were found to be 25% and 9%, respectively. Both prevalence rates represented a lower percentage compared to prevalence rates identified in other European countries participating in the survey (30% and 13%, respectively) [9]. However, the frequency of particular nutritional risk factors in hospitalized patients, i.e., unintended body weight loss or insufficient recent food intake, was approximately two times higher than the frequency of malnutrition risk both in Polish and in the whole European study population. The study also revealed that managing patients with malnutrition or risk of malnutrition in Polish hospital wards is inferior to that carried out in European facilities involved in the survey. For example, it showed four times lower commitment of nutrition experts in managing patients with identified malnutrition or risk of malnutrition. This may result in noticed lower supply of special diets (8% vs 16%) and oral nutritional supplements (4% vs 12%) in Polish hospital wards [9]. One of the measures to assist in the elaboration of a comprehensive concept of nutritional care for patients could be the implementation of tools that integrate the process of identification of malnourished patients with the decision-making pathway for the implementation of appropriate management. A tool that addresses not only screening and assessment but also monitoring and triaging for interventions is the Patient-Generated Subjective Global Assessment (PG-SGA) [10].

The PG-SGA adequately covers all domains of the construct of malnutrition [5]. The PG-SGA was designed as a two-page form that is fitted with a scoring system. The first page is patient-generated, including four boxes that address patients’ weight, food intake, symptoms affecting food intake (i.e., nutrition impact symptoms), and level of activity and functioning. The latter two components extend beyond the GLIM criteria and broaden the assessment of phenotypic and etiologic hallmarks of malnutrition with relevant inputs for triaging for interventions [11]. The second page is a professional component consisting of five worksheets designed to score weight loss, patients’ diagnosis in relation to nutritional requirements, metabolic stress, and body composition (muscle, fat, and fluid status), and, finally, to summarize the total PG-SGA with its conversion in nutritional triage recommendation and patient’s global assessment stage categorization. Combining patient- and professional-generated data allows complex insights into the nutritional status of patients, which makes PG-SGA a unique tool for use in nutritional care. While used most widely in oncology, the tool was originally developed to be agnostic to the underlying cause of the malnutrition or nutritional risk.

The PG-SGA has been shown to be more sensitive and specific in the nutritional assessment of cancer patients than other tools [12] and is often considered a semi-gold standard for nutritional screening in oncology [13, 14]. The validity of PG-SGA has also been confirmed in evaluating the nutritional status of nephrology and neurology patients, in elderly and palliative care, among others [15–18]. The PG-SGA has already been culturally adapted and linguistically validated for use in various settings worldwide, including Portuguese, Dutch, Thai, German, and more recently Norwegian, Greek, and Japanese, as well as Danish and Italian [19–27]. The development of these new PG-SGA language versions was based on the Principles of Good Practice for the Translation and Cultural Adaptation Process (ISPOR) [28]. So far, no validated Polish version of the PG-SGA is available. Therefore, we aimed to translate and culturally adapt the original English PG-SGA for the Polish setting, and to assess the linguistic validity as perceived by the Polish population, both patients and healthcare professionals (HCPs), and content validity by HCPs only.

## Methods

### Translation and cultural adaptation

The translation and cultural adaptation process of the PG-SGA for the Polish setting has been carried out with permission and in close cooperation with the key developer and copyright holder of the PG-SGA (FO) and an international expert on translation and cultural adaptation of the PG-SGA (HJ-W).

The entire process of translation, cultural adaptation, and content and linguistic validation was conducted between August 2016 and January 2021, according to the International Society for Pharmacoeconomics and Outcomes Research (ISPOR) guideline “Translation and Cultural Adaptation of Patient-Reported Outcomes Measures—Principles of Good Practice” (ISPOR principles) [28]. The ISPOR principles consist of the ten steps in the translation and cultural adaptation process:

- Step 1. Preparation: The project manager made contact with the key developer of the original version of the PG-SGA and the international expert on translation and cultural adaptation of the PG-SGA.
- Step 2. Forward translation: This step was carried out by three native Polish speakers (the project manager/key in-country consultant and two project co-managers) who are experts on the subject of nutritional assessment and with sufficient knowledge of the English language.
- Step 3. Reconciliation: The independent three forward translations were compared with each other and transferred into one integrated forward translation (Polish PG-SGA version 1) that has been subject to verification by an independent group of experts in terms of cultural and semantic content. The group of experts (*n* = 20) included dietitians (*n* = 5), physicians (*n* = 8), nurses (*n* = 2), a pharmacist (*n* = 1), a psychologist (*n* = 3), and a physiotherapist (*n* = 1). After consensus was reached, the reconciled forward translation, i.e., Polish PG-SGA version 2 (Polish PG-SGA v2), was finalized.
- Step 4. Back translation: Two back translations of the reconciled forward translation were independently carried out by two certificated translation companies.
- Steps 5 and 6. Back translation review and harmonization: All differences between the original English version and the two back translations were discussed at a meeting of the project manager/key in-country consultant and two project co-managers, the developer of the PG-SGA, and the international expert on cross-cultural adaptation of the PG-SGA. The Polish PG-SGA v2 was adapted, if needed, to harmonize with the original English version.

### Linguistic and content validation

The linguistic and content validation of PG-SGA Polish v2 included steps 7–9 of the ISPOR guidelines. Step 7 concerns cognitive debriefing of the Polish translation in a heterogeneous group of patients and HCPs. The purpose of this step is to ensure that the translation is comprehensible and easy to complete by general population of patients and HCPs and that the HCPs perceive the Polish PG-SGA as relevant.

- Step 7. Cognitive debriefing: This step comprises cognitive debriefing of the Polish PG-SGA v2 with patients and HCP groups.

Three components were used to evaluate the items in the Polish PG-SGA v2:

- Comprehensibility: How clearly is the item described in the tool?
- Difficulty: How difficult is it to answer the item (does the respondent need more knowledge or skills to be able to answer the item)?
- Content validity: Does the respondent consider the item relevant to the concept of malnutrition?

### Patient group

For the cognitive debriefing, 174 patients from various regions of Poland evaluated comprehensibility and difficulty of the PG-SGA patient component, i.e., the Polish PG-SGA Short Form version 2 (Polish PG-SGA SF v2). The adult, Polish native speaking participants were recruited between May 2018 and December 2020, from Lower-Silesian Oncology Center in Wroclaw, Uzdrowisko Wysowa S.A. dialysis station (SPA Hospital) in Wysowa Zdrój and University Clinical Hospital in Wroclaw (Table 1). The comprehensibility and difficulty evaluation was carried out using a questionnaire. The questionnaire contained demographic information and 36 questions addressing comprehensibility, six addressing difficulty, and four open-ended questions with feedback on the Polish wordings. The degree of difficulty and comprehensibility was assessed by a 4-point Likert scale. The questionnaire was used before in other studies concerning cultural adaptation and validation of the PG-SGA [19–27].

**Table 1:** Characteristics of the patient participants (*n* = 174)

Variables	
Gender, % (*n*)^a^	
Female	52 (74)
Age [years], mean (± SD)	56 (± 16.1)
Education level % (*n*)	
Primary school	6 (11)
Secondary school	49 (84)
Higher education	35 (60)
Basic vocational school	10 (18)
Diagnosis, % (*n*)	
Kidney failure	34 (59)
CVD	28 (49)
Cancer	16 (28)
Other	22 (38)

*CVD*, cardiovascular disease.

^a^Stated by 143 patients.

### Healthcare professionals

In parallel, 188 HCPs participated in evaluating comprehensibility, difficulty, and content validity of the full Polish PG-SGA v2. This group of participants consisted of physicians, nurses, dietitians, nutritionists, and medical students recruited from hospitals and primary health care clinics from various regions of Poland, Lower-Silesian Oncology Center in Wroclaw, Uzdrowisko Wysowa S.A. dialysis station (SPA Hospital) in Wysowa Zdrój, University Clinical Hospital in Wroclaw, Wroclaw Medical University, and University of Applied Sciences in Nysa (Table 2). They were asked to provide demographic information (16 questions) and to complete a questionnaire consisting of 38 questions on comprehensibility and 38 on difficulty of the professional component of the PG-SGA (i.e., the Worksheets), and 75 on content validity of the full version PG-SGA. Additionally, the respondents were asked to complete eight open-ended questions for feedback on the Polish wordings of the professional component of the PG-SGA. The degree of difficulty, comprehensibility, and content validity was assessed by a 4-point Likert scale. The questionnaire has previously been used before in other studies concerning cultural adaptation and validation of the PG-SGA [19–27].

**Table 2:** Characteristics of HCP participants (*n* = 188)

Variables	
Current profession, % (*n*)	
Nutritionist	5 (9)
Dietitian	5 (9)
Physician	42 (78)
Nurse	25.0 (47)
Student, dietetics	5 (10)
Student, medicine	4 (7)
Student, pharmacy	11 (21)
Student, nursing	3 (6)
Other	1 (1)
Place of employment, % (*n*)	
Hospital	22 (42)
Private practice	9 (17)
Primary healthcare	16 (31)
University	50 (94)
Years of experience in the current profession^a^, median (min–max)	8 (0.5–41)
Caring for the patient population, % (*n*)	
Oncological	20 (37)
Nephrological	28 (52)
Surgical	7 (13)
Geriatric	24 (45)
Other	35 (66)
Familiar with PG-SGA—yes, % (*n*)	19 (36)
Experience with PG-SGA—yes, % (*n*)	6 (12)

^a^Stated by 143 professionals

The Wroclaw Medical University Bioethics Committee (KB-540/2019) approved the study. The study was conducted in accordance with the Declaration of Helsinki. Each participant (patient and HCP) provided informed consent before participating in the study after reading the consent form of participation in the study:

This is the study on the validation of the translated and culturally adapted PG-SGA form for the Polish setting. Completing the survey will take you approximately 20 min and is completely anonymous. The study aims to assess comprehensibility and difficulty of PG-SGA Short Form by patients, and additionally content validity, comprehensibility and difficulty of Short Form and professional component by HCPs. Comprehensibility means how clearly is the item described in the tool. Difficulty—how difficult is to answer the item (e.g. do you need more knowledge or skills to be able to answer the item). Content validity answers the question if you consider the item relevant to the concept of malnutrition. Evaluations are operationalized by a 4-point scale. If you do not wish to continue with the survey you can stop answering the questions on the form at any time.

- Step 8. Review of cognitive debriefing and finalization: The results of the questionnaire posed to patients and HCPs were reviewed by the project manager and co-managers as well as the developer of the PG-SGA, and the international expert on translation and cultural adaptation of the PG-SGA. Based on the respondents’ suggestions, necessary changes to improve the translated items were discussed with and approved by the developer of the PG-SGA. After review, the Polish version of the PG-SGA was finalized by the project manager and co-managers.
- Step 9. Proofreading: The finalized Polish version of the PG-SGA was proofread by the project manager and co-managers to check for possible grammar and spelling errors, as well as the legibility of the graphic form.
- Step 10. Final report: The final report includes a description of all stages of validation and cultural adaptation of the Polish version of PG-SGA and conclusions drawn on the basis of the obtained validation results.

### Statistical analysis

For item and scale scores calculations, scores of 1 and 2 were recoded into 0, as “not present,” while scores of 3 and 4 were recoded into 1, indicating “present” [20]. Item indices were calculated by dividing the number of respondents who considered the item to be “present” by the total number of respondents. Indices were calculated for each item for comprehensibility (I-CI), difficulty (I-DI), and content validity (I-CVI). Item indices < 0.78 required further analysis of the item.

The scale indices were calculated by averaging all item indices for the respective construct: for comprehensibility as Scale Comprehensibility Index (S-CI), for difficulty as Scale Difficulty Index (S-DI), and for content validity as Scale Content Validity Index (S-CVI). Patients evaluated comprehensibility and difficulty of the patient component of the PG-SGA only; and therefore, the patient scale indices (S-CI and S-DI) are only referring to the PG-SGA Short Form. HCPs evaluated content validity of the PG-SGA Short Form and of the professional component of the PG-SGA, resulting in a total scale index for the full PG-SGA. Comprehensibility and difficulty were evaluated by HCPs only, for the professional component of the PG-SGA. Scale indices 0.80–0.89 were considered acceptable and ≥ 0.90 as excellent.

All calculations of the item indices and index scores were performed in Excel. Continuous variables are presented as means and standard deviation (SD) or medians and range. Categorical variables are presented as frequencies (number) and percentage. The Chi^2^ test was used for distribution comparison of categorical variables between groups. The independent sample *t*-test (for parametric data) or Mann–Whitney *U* test (for non-parametric data) was used to analyze differences between two groups, while one-way ANOVA or Kruskal–Wallis test was used for more than two groups. Post hoc analyses were performed for an intergroup comparison of data. All statistical analyses were performed using STATISTICA v. 13.3 (StatSoft, Inc., Tulsa, USA). A *p*-value < 0.05 was considered to indicate statistically significant differences.

## Results

### Steps 1–6

During the forward translation process, in which three independent Polish translations of PG-SGA were produced, 36/55 items of the patient component and 66/97 items of the professional component differed between each other. The reconciliation step (data are available upon request to the corresponding author) resulted in the Polish PG-SGA v1. Then, back translations provided by two independent persons revealed additional 30 discrepancies after review against the original version, which were further discussed with the developer of the original version of the PG-SGA and the international expert on translation and cultural adaptation of the PG-SGA (data can be made available upon request to the corresponding author). After harmonization, the Polish PG-SGA v2 was then included into cognitive debriefing and exploration of content validity by questionnaires administered to patients and HCPs.

### Step 7

To ensure inclusion of a heterogeneous population of patients, 174 patients with different diagnoses, including kidney failure (34%), cardiovascular diseases (CVD) (28%), different cancers (16%), and other diseases, completed the evaluation of comprehensibility and difficulty of the Polish PG-SGA SF v.2. Detailed characteristics of the participating patients are presented in Table 1.

Among 188 HCPs who completed the questionnaire developed for specialists, 78 were physicians, 47 nurses, 45 students of different faculties (pharmacy, dietetics, medicine, and nursing), and 18 dietitians/nutritionists. The median time of experience within their respective fields was 8.0 (range 0.5–41.0) years. From the total group of HCPs, 19% were familiar with the PG-SGA, mainly from literature, courses, or other sources (e.g., their own research). However, only 6% of all professionals had experience with PG-SGA. Detailed characteristics of the participating HCPs are presented in Table 2.

The indices of comprehensibility, difficulty (as perceived by patients), and content validity (as perceived by HCPs for the Polish PG-SGA SF v2) are presented in Table 3. Comprehensibility of the patient component of the PG-SGA was perceived as excellent by the patient group, with S-CI = 0.96 and individual item scores ranging from 0.90 to 1.00. Scores given by patients on difficulty fell into the excellent range (S-DI = 0.94) with individual item scores ranging from 0.93 to 0.95. Content validity for the patient component as perceived by the HCPs was excellent, with S-CVI = 0.90. No item indices were below acceptable range (< 0.80).

**Table 3:** Indices for comprehensibility, difficulty, and content validity for the patient component of the Polish Patient-Generated Subjective Global Assessment

Item	Patients				HCPs	
	*n*	CI	*n*	DI	*n*	CVI
Box 1: Weight						
1a I currently weigh about _kg	173	0.99	171	0.95	188	0.97
1b I am about _cm tall	173	0.99			187	0.95
1c One month ago, I weighed about _kg	170	1.00			186	0.94
1d Six months ago, I weighed about _kg	171	0.99			187	0.94
1e Weight (decreased/not changed/increased)	171	0.96	167	0.95	183	0.93
Box 2: Food intake						
2a As compared to my normal intake, I would rate my food intake during the past months as	162	0.96	173	0.95	185	0.97
2a1 Unchanged/more than usual/less than usual	171	0.96			187	0.93
2b I am now taking	166	0.95	172	0.93	176	0.85
2b1 Normal food, but less than normal amount	169	0.96			186	0.83
2b2 Little solid foods	171	0.91			186	0.80
2b3 Only liquids	169	0.94			186	0.88
2b4 Only nutritional supplements	170	0.91			186	0.87
2b5 Very little of anything	171	0.90			185	0.86
2b6 Only tube feeding or nutrition by vein	171	0.94			185	0.92
Box 3: Symptoms			172	0.93		
3a. I have the following problems that have kept me from eating enough during the past 2 weeks	155	0.96			187	0.95
3a1 No problems eating	173	0.98			186	0.89
3a2 No appetite. Just did not feel like eating	170	0.96			187	0.95
3a3 Vomiting	170	0.97			186	0.95
3a4 Nausea	170	0.95			187	0.92
3a5 Diarrhea	170	0.97			187	0.96
3a6 Constipation	171	0.95			187	0.91
3a7 Dry mouth	169	0.97			186	0.88
3a8 Mouth sores	170	0.96			187	0.94
3a9 Smells bother me	171	0.96			187	0.82
3a10Things taste funny or have no taste	170	0.94			187	0.86
3a11 Feel full quickly	173	0.97			187	0.88
3a12 Problems swallowing	170	0.97			187	0.95
3a13 Fatigue	173	0.96			187	0.81
3a14 Pain: where?____________	169	0.98			186	0.95
3a15 Other	169	0.96			163	0.82
Box 4: Activities and Function			172	0.94		
4a Over the past month I would generally rate my activity as	171	0.95			186	0.89
4a1 Normal with no limitation	170	0.95			185	0.86
4a2 Not my normal self, but able to be up and about with fairly	171	0.96			184	0.88
4a3 Not feeling up to most things, but in bed of chair less than half the day	171	0.95			184	0.91
4a4 Able to do little activity and spend most of the day in bed or chair	169	0.95			184	0.91
4a5 Pretty much bedridden, rarely up of bed	169	0.96			181	0.92
Scale Indices patient component	5860	0.96	1027	0.94	6661	0.90

*CVI*, Content Validity Indices; *CI*, Comprehensibility Indices; *DI*, Difficulty Indices.

Further analyses indicated that scales of comprehensibility and difficulty were significantly different among patients with different levels of education (Table 4). The highest S-CVI was perceived by patients with the highest education level (university). On the other hand, patients with secondary school evaluated difficulty at the lowest rate among all participants. Education level did not disclose scores of comprehensibility and difficulty below excellent range (> 0.90).

**Table 4:** Comprehensibility and difficulty of patient component Polish version of PG-SGA per level of patient education and age

	Scale CI	*p*-value	Scale DI	*p*-value
Level of education				
Primary school	0.95^ab^	0.014	0.98^a^	0.025
Secondary school	0.95^b^		0.95^ab^	
Vocational school	0.94^ab^		0.85^b^	
University	0.98^a^		0.96^a^	
Age				
≤ 65 years	0.96	0.121	0.94	0.689
> 65 years	0.96		0.94	

Differences were evaluated using Mann–Whitney *U* test (*p* < 0.05); the values in the same column that share the same letter are not significantly different.

*CI*, Comprehensibility Indexes; *DI*, Difficulty Indexes.

Table 5 presents comprehensibility, difficulty, and content validity of the professional component of the Polish PG-SGA v2 as perceived by HCPs. Overall, comprehensibility, difficulty, and content validity of worksheets were perceived as acceptable (S-CI = 0.87, SDI = 0.80, and S-VI = 0.86, respectively). In detail, comprehensibility and difficulty of 10/38 and 6/38 items, respectively, were perceived as below acceptable range (< 0.80). Moreover, content validity of 14/34 items fell below the predefined cutoff of 0.80. For comprehensibility and difficulty, all item scores that fell below cutoff for acceptability were from Worksheet 4 (*Physical exam*). For content validity, item scores below cutoff were from Worksheet 3 (*Metabolic demand*) and Worksheet 4 (*Physical exam*).

**Table 5:** Indices for content validity, comprehensibility, and difficulty for the professional component of the Polish Patient-Generated Subjective Global Assessment

Item	*n*	CVI	*n*	CI	*N*	DI
Worksheet 1	173	0.92	183	0.92	178	0.84
Scoring weight (Wt) loss	181	0.91	177	0.90	174	0.84
Worksheet 2. Disease and its relations to nutritional requirements	182	0.96	166	0.93	178	0.93
2a Cancer	184	0.96	184	0.93	185	0.90
2b. AIDS	184	0.94	184	0.95	185	0.88
2c. Pulmonary or cardiac cachexia	186	0.95	184	0.96	185	0.90
2d. Chronic renal insufficiency	186	0.96	184	0.96	185	0.91
2e. Presence of decubitus, open wound or fistula	186	0.94	184	0.96	184	0.91
2f. Presence of trauma	186	0.90	184	0.91	184	0.88
2 g. Age greater than 65	185	0.88	184	0.95	183	0.91
2 h. All relevant diagnoses	176	0.91	184	0.89	182	0.85
2i. Primary disease staging (circle if known or appropriate) I, II, III, IV, other	179	0.83	182	0.81		
Worksheet 3. Metabolic demand	156	0.88	169	0.83	173	0.81
3a. Fever	185	0.88	184	0.88	184	0.85
3b. Fever duration	185	0.88	184	0.89	184	0.79
3c. Corticosteroids	185	0.88	183	0.86	183	0.79
Worksheet 4. Physical exam	161	0.88	172	0.80	168	0.70
4a. Temples	184	0.73	182	0.83	182	0.64
4b. Clavicles	187	0.66	185	0.79	184	0.67
4c. Shoulders (deltoids)	187	0.74	185	0.79	184	0.73
4d. (Interosseous muscles)	187	0.67	184	0.73	184	0.62
4e. Scapula (latissimus dorsi, trapezius, deltoids)	187	0.68	185	0.78	184	0.68
4f. Thigh (quadriceps)	187	0.78	185	0.84	184	0.78
4 g. Calf (gastrocnemius)	187	0.76	185	0.83	184	0.80
4 h. Global muscle status rating	185	0.87	184	0.86	183	0.77
4i. Orbital fat pads	185	0.62	185	0.86	184	0.54
4j. Triceps skinfold	185	0.75	185	0.78	184	0.72
4 k Fat overlying lower ribs	186	0.70	185	0.79	183	0.69
4 l. Global fat deficit rate	184	0.88	184	0.86	183	0.77
4 m. Ankle edema	186	0.92	185	0.92	184	0.85
4n Sacral edema	186	0.82	185	0.87	184	0.81
4o. Ascites	185	0.93	185	0.90	184	0.81
4p. Global fluid status rating	186	0.92	185	0.90	184	0.80
Worksheet 5. Global Assessment Categories Stage A: well nourished; Stage B: moderate/suspected malnutrition; Stage C: severely malnourished	175	0.94	180	0.93	181	0.86
Nutritional triage recommendations:	181	0.93	180	0.89	183	0.83
Triage: 0–1, no intervention required at this time. Re-assessment on routine and regular basis during treatment	185	0.89	184	0.93		
Triage: 2–3, patient and family education by dietitian, nurse, or other clinician with pharmacologic intervention as indicated by symptom survey (box 3) and lab values as appropriate	184	0.89	184	0.91		
Triage: 4–8, requires intervention by dietitian, in conjunction with nurse or physician as indicated by symptoms (box 3)	185	0.93	184	0.92		
Triage ≥ 9, indicates a critical need for improved symptom management and/or nutrient intervention options	185	0.92	184	0.93		
Scale Indices professionals component	7129	0.86	7120	0.87	6380	0.80
Scale Indices full PG-SGA	11,904	0.88				

Items below acceptable range (< 0.78) are marked in bold.

*CVI*, Content Validity Indices; *CI*, Comprehensibility Indices; *DI*, Difficulty Indices.

We found significant differences in all (content validity, comprehensibility, and difficulty) scale indices (*p* < 0.05 for all) between HCPs with different professions, as well as in S-CVI and S-CI between HCPs being familiar with PG-SGA vs those who being not. Those HCPs who were familiar with PG-SGA perceived the Polish PG-SGA v2 as less comprehensible and with lower content validity than those who were unfamiliar (Table 6).

**Table 6:** Content validity, comprehensibility, and difficulty of Polish version per profession, prior experience, and familiarity with PG-SGA

	Scale-CVI	*p*-value	Scale-CI	*p*-value	Scale-DI	*p*-value
Profession						
Physicians	0.83^c^	< 0.01	0.83^b^	< 0.01	0.75^b^	< 0.01
Nurses	0.91^a^		0.86^ab^		0.75^b^	
Dietitians	0.95^b^		0.96^c^		0.93^a^	
Students	0.90^a^		0.91^a^		0.87^a^	
Experience						
Yes	0.88	0.18	0.88	0.71	0.77	0.66
No	0.88		0.87		0.80	
Familiarity						
Yes	0.85	< 0.01	0.84	0.03	0.77	0.14
No	0.89		0.88		0.81	

Items below acceptable range (< 0.78) are marked in bold; differences were evaluated using Mann–Whitney *U* test (*p* < 0.05); the values in the same column that share the same letter are not significantly different.

*CVI*, Content Validity Indexes; *CI*, Comprehensibility Indexes; *DI*, Difficulty indexes.

Table 7 presents significant differences in the evaluation of content validity, comprehensibility, and difficulty of particular items among HCPs. Physicians followed by nurses rated particular items at the lowest score of content validity, comprehensibility, and difficulty. For content validity, most differences among HCPs were observed in Worksheet 4 (*Physical exam*), with the lowest scores among physicians and nurses. For comprehensibility, differences in scores between HCPs also concerned items mainly related to Worksheet 4 (*Physical exam*), with the lowest percentages of a high rating from physicians and nurses. Additionally, Worksheet 3 (*Metabolic demand*) and Worksheet 4 (*Physical exam*) were perceived as difficult also mostly by physicians and nurses. The difficulty of some items of Nutritional Triage Recommendations was evaluated as below the cutoff for acceptability only by physicians, presenting significantly higher percentages of a low rating for these items in comparison with other specialists.

**Table 7:** Significant differences between groups of HCPs in content validity, comprehensibility, and difficulty evaluation

Items	Students	Nurses	Dietitians/nutritionists	Physicians	Pearson Chi^2^ (*p*-value)
Relevance/content validity					
Smells bother me	0.83	0.91	0.94	0.72	0.035
Fatigue	0.80	0.96	0.88	0.71	0.010
Scoring weight loss	0.93	0.98	1.00	0.85	0.048
Age greater than 65	0.94	0.96	1.00	0.79	0.005
All relevant diagnoses	0.98	0.95	1.00	0.84	0.009
Primary disease staging (circle if known or appropriate) I, II, III, IV, other	0.86	0.98	0.94	0.67	0.0002
Worksheet 3—metabolic demand	0.95	0.97	0.93	0.77	0.003
Corticosteroids	0.91	0.98	0.88	0.81	0.021
Clavicles (pectoralis and deltoids)	0.85	0.73	0.82	0.47	< 0.001
Shoulders (deltoids)	0.85	0.78	0.88	0.62	0.012
Interosseous muscles	0.83	0.78	0.82	0.48	< 0.001
Scapula (latissimus dorsi, trapezius, deltoids)	0.83	0.71	0.88	0.53	< 0.001
Orbital fat pads	0.78	0.67	0.82	0.45	< 0.001
Triceps skin fold	0.89	0.73	1.00	0.61	< 0.001
Fat overlying lower ribs	0.78	0.76	1.00	0.55	< 0.001
Sacral edema	0.81	0.96	0.94	0.72	0.003
Comprehensibility					
Fever	0.96	0.80	1.00	0.85	0.044
Clavicles (pectoralis and deltoids)	0.91	0.78	0.88	0.70	0.030
Scapula (latissimus dorsi, trapezius, deltoids)	0.91	0.71	0.88	0.71	0.028
Calf (gastrocnemius)	0.93	0.73	0.94	0.79	0.033
Orbital fat pads	0.83	0.73	0.76	0.54	0.006
Difficulty					
Pulmonary or cardiac cachexia	0.98	0.86	1.00	0.84	0.044
Metabolic demand	0.89	0.73	1.00	0.77	0.049
Fever	0.96	0.73	0.94	0.84	0.014
Corticosteroids	0.91	0.72	1.00	0.82	0.027
Clavicles (pectoralis and deltoids)	0.80	0.64	0.82	0.56	0.021
Interosseous muscles	0.78	0.60	0.65	0.52	0.038
Orbital fat pads	0.74	0.51	0.59	0.43	0.009
Triceps skin fold	0.85	0.60	0.88	0.68	0.020
Global fat deficit rating	0.87	0.60	0.94	0.76	0.006
Ankle edema	0.89	0.71	1.00	0.88	0.011
Global fluid status rating	0.91	0.67	1.00	0.77	0.007
Nutritional triage recommendations:	0.93	0.80	1.00	0.75	0.013
0–1	0.91	0.80	1.00	0.75	0.026
2–3	0.91	0.80	1.00	0.75	0.026
4–8	0.91	0.80	1.00	0.75	0.026
≥ 9	0.89	0.80	1.00	0.75	0.046

Items below acceptable range (< 0.78) are marked in bold.

### Steps 8–10

After cognitive debriefing, in consideration of the individual comments given by the respondents to particular items, the key country members, the developer of the PG-SGA, and the international expert on translation and cultural adaptation of the PG-SGA consulted together and agreed to make some changes to the final Polish PG-SGA version 3 (Polish PG-SGA v3). These changes improved items in clarification and for better adaptation to the Polish settings (Table 8). For instance, in Box 2 about Food intake, the Polish answer item “very little of anything” was replaced by “very small amounts of any food” and “solid foods” was changed to “foods with solid consistency,” while in Box 3, the phrase “eating enough” was changed to “adequate amounts of food.” Additionally, in the professional component, in Worksheet 1, we decided to bold weight loss in 1 month, because HCPs were often confused about which information on weight loss they should use (1 month vs 6 months). All comments and suggestions from the patients and HCPs were taken into account and individually considered in finalizing the culturally adapted and linguistically validated version of the Polish Scored PG-SGA.

**Table 8:** Changes of expression most often raised by patients and HCPs (PG-SGA Polish version) after review of cognitive debriefing results and finalization steps

	Before	After	Reason
Box	Patients		
B1	masa ciała (Eng. body weight)	waga (eng. body weight)	The word “waga” is more common and used in colloquial language, while “masa ciała” is more specific, often used by specialists
B2	Bardzo małe ilości czegokolwiek (Eng. very little of anything)	Bardzo małe ilości jakichkolwiek pokarmów (eng. very small amounts of any food)	It sounds better in Polish
B2	Preparaty odżywcze (Eng. nutritional supplements)	Preparaty odżywcze odżywki (np. nutridrinki, odżywki) (eng. nutritional supplements (e.g., nutridrinks, supplements)	Examples of nutritional supplements were added, because some patients were confused what “preparaty odżywcze” are
B2	Pokarmy stałe (Eng. solid foods)	Pokarmy o stałej konsystencji (eng. foods with solid consistency)	The word “konsystencja” was added because in Polish “stały” means also (except of solid) that something is done routinely, constantly
B3	wystarczających ilości pokarmów (Eng. eating enough)	odpowiednich ilości pokarmów (eng. adequate amounts of food)	It sounds better in Polish
B4	Normalną bez ograniczeń (Eng. normal, with no limitations)	Taką jak zwykle, bez ograniczeń (eng. as usual, with no limitations)	The word “normalna” was changed into “taka jak zwykle,” because patients were confused what normal physical activity means. The phrase: physical activity as usual relates more to the comparison between before and currently, than to the so-called norm
Worksheet	HCPs		
W1	Weight loss in 1 month vs weight loss in 6 months	We decided to bold weight loss in 1 month	HCPs were confused which weight loss should they use (1 month vs 6 months)
W2	Lack of semicolon between I and II clinical stage	Semicolon inserted	Editorial error
W3	72 godziny (Eng. 72 h)	72 godziny (3 dni) (eng. 72 h (3 days))	The HCPs had a problem with which accuracy to determine the duration of fever (e.g., whether 71.5 h is still < 3 days). The addition of the unit “day” makes it easier to relate the duration of the fever to the needs of the sheet

## Discussion

Translation and cultural adaptation of the original PG-SGA (©2005) according to ISPOR Principles resulted in the Polish version which has preserved the purpose and conceptual meaning of the original PG-SGA. Independent forward and back translations, reconciliation, and harmonization with the key developer and the international expert on translation and cultural adaptation of the PG-SGA and further cognitive debriefing with a large group of heterogeneous patients and HCPs delivered another new language version of the PG-SGA with conceptual and cognitive equivalence to the original English PG-SGA.

Linguistic and content validation revealed that the patient component of the Polish version of the PG-SGA was very well comprehensible and easy to complete for the patient. Obtained results indicated that the Polish version of the patient component, i.e., PG-SGA Short Form, can be further completed without any additional instruction from HCPs. Moreover, HCPs indicated that the patient component of the Polish version of the PG-SGA was relevant. Similar results on the patient component were obtained in other studies performed in Portugal [19], the Netherlands [20], Thailand [21], Germany [22], Norway [23], Greece [24], Japan [25], Denmark [26], and Italy [27]. In all mentioned studies, content validity, as well as comprehensibility and difficulty of the PG-SGA Short Form, was perceived as excellent. This indicated that the translated and culturally adapted PG-SGA Short Form is considered feasible to perform by patients in various countries and/or cultures.

Our results revealed that comprehensibility and difficulty of the PG-SGA Short Form did not differ between older (age > 65 years) or younger adult patients. This indicates that the Polish version of the PG-SGA Short Form is understandable and easy to complete for both younger and older patients. However, the level of patients’ education had an impact on the results. Perceived comprehensibility was best by patients who graduated from university, followed by those who graduated from primary, secondary, and vocational school. Most patients perceived the PG-SGA Short Form as very easy to complete (i.e., excellent difficulty), with the exception of those with a vocational education, who found the difficulty acceptable. In contrast, the mean indices for difficulty were the highest in patients with primary education. It should be noted that the representation of patients with these two levels of education was the lowest (respectively 6% and 10%) in the entire recruited group, and concluding about the reasons for such a distribution of results is not possible. In the Japanese study, the relationship between educational level and difficulty was also analyzed, and in that study, no relationship between education level and difficulty of the PG-SGA Short Form as perceived by patients was found [25].

Comprehensibility and difficulty of the professional component of the Polish version of the PG-SGA were considered acceptable by HCPs (S-CI = 0.87 and S-DI = 0.80, respectively). These results are in contrast to the results of the Dutch [20], Thai [21], German [22], Norwegian [23], and Japanese [25] studies, in which the professional component of the PG-SGA scored below acceptance in the constructs of difficulty, and additionally of comprehensibility in the Norwegian study [24]. On the other hand, in a recently published study on the validation of the Greek version of the PG-SGA [24], comprehensibility and difficulty of the professional component were perceived as excellent (≥ 0.90). Also in the Italian study, comprehensibility, as well as content validity, was perceived as excellent, but difficulty was considered acceptable by professionals [27]. Despite presented differences in comprehensibility and difficulty, relevance of the professional component of the PG-SGA was perceived as excellent in almost all performed studies on validation of translations and cultural adaptations of the PG-SGA [22, 23]. Only results of our study and the Dutch study [20] indicated that professionals perceived content validity of the PG-SGA as acceptable. Additionally, content validity of the Full PG-SGA was perceived in the current and other studies as acceptable [20] or even excellent [22, 23]. Lower ratings on relevance obtained in our study and the Dutch study [20] came mainly from low ratings of the *Physical exam* in *Worksheet 4.*

In line with other studies on linguistic and content validation of the PG-SGA, *Worksheet 4 (Physical exam)* was considered the most difficult and most incomprehensible, and with the lowest scores on content validity from all worksheets of the professional component of the PG-SGA. Difficulty considered by HCPs was below acceptance for about 70% of items from this worksheet, and these items concerned mainly evaluation of muscle status. Additionally, HCPs evaluated that comprehensibility of about one-third of all items from *Worksheet 4*, i.e., the physical exam, was below acceptable range. Most importantly, the content validity of almost 60% of all items from this worksheet was considered unacceptable. This indicated that HCPs seem to feel no need to perform a physical exam, especially so detailed for muscle status rating. It is unclear why the relevance of the physical exam was rated low by the Polish HCPs, especially since HCPs already familiar with the PG-SGA perceived less comprehensibility and relevance, but we speculate that insufficient knowledge about how to apply the PG-SGA may play a role. In other words, familiarity with the tool does not guarantee its comprehension. For example, it is not always known that the inclusion of seven muscle groups in Worksheet 4 does not mean that all seven muscle groups need to be evaluated in every patient. Previous studies have demonstrated that a 1-day training on the PG-SGA increases perceived comprehensibility and difficulty. In the Dutch study performed in dietitians [29], difficulty and comprehensibility indices related to muscle status in Worksheet 4 increased from 0.16 to 0.54 and from 0.41 to 0.91 after the training, respectively. Also, in the Portuguese study [30] that was performed in a sample of mostly dietitians as well, training in the PG-SGA improved ratings on difficulty, mostly concerning items of *Physical exam*. In that study, the maximum difference in scale index scores on the difficulty of particular items related to the physical exam was 0.258. These studies [29, 30] demonstrate high effectiveness of training, in which knowledge and skills on applying the PG-SGA are expanded, on perceived difficulty, and comprehensibility especially among HCPs who had no experience with this questionnaire. We hypothesize that higher scores on difficulty and comprehensibility may in turn result in improved perceived relevance of the physical exam of the PG-SGA. Additional advantages of training could be more reliable results.

A detailed analysis of results among different HCPs revealed that physicians evaluated comprehensibility, difficulty, and content validity lowest, while difficulty was even below acceptable threshold. These results would indicate that physicians were more conscious of a possible lack of experience, especially in the term of muscle status rating. However, in the Norwegian study [23], no significant differences between type of HCP or familiarity with the PG-SGA were found. In our study, only 6% of all participants had actually been experienced with the PG-SGA before, and the familiarity with PG-SGA was reported by 19% of HCPs and came mainly from the literature. Additionally, in our study, we found a significant difference in comprehensibility and content validity depending on the knowledge of the tool. Surprisingly, HCPs who had prior knowledge on the PG-SGA evaluated content validity and comprehensibility lower than those who were not familiar with PG-SGA. These results indicate that training in the use of PG-SGA before daily practice is needed for everyone, including those already familiar with PG-SGA from the literature. Theoretical knowledge did not translate into higher content validity, i.e., perceived relevance.

### Implications for practice and research

Nutritional screening tools are used to identify patients at nutritional risk. To be useful, a tool must be practical, easy to carry out, cost-effective, not time-consuming, highly sensitive, and have good specificity, and be highly reliable, that means having a small variation between observers and separate time points. All these conditions are met by the PG-SGA Short Form [31, 32], while the Full PG-SGA is a tool for nutritional assessment, triage, and monitoring. It has demonstrated good sensitivity, specificity, and positive and negative predictive value, as well as very good predictive value for overall survival and quality of life in patients at risk of malnutrition [32–34].

The use of ISPOR Principles during the translation and cultural adaptation of the original English version of PG-SGA into other languages ensures that the original purpose and intention of the key developer will be preserved and will ensure the cultural equivalence of the tool. So far, the PG-SGA has been translated and culturally adapted into 11 languages and linguistically validated in this way, and more language versions similarly produced are being developed [35]. The results for the Polish translation and cultural adaptation indicate that the Polish PG-SGA version maintained purpose, meaning, and format equivalent to the original PG-SGA. Furthermore, the acceptable values for content validity reflect that the Polish version presented in this paper is ready for use in clinical practice and in future studies conducted in the Polish language. In connection with the above, the PG-SGA can be easily implemented in the Polish language, and scientific and clinical results will be reliable and consistent with international data derived from validated versions of this tool. Nevertheless, before implementing the full PG-SGA in clinical practice, training of professionals is recommended.

### Strengths and limitations of the study

A major strength of the current study is the application of the ISPOR Principles, similar to the previous studies on new language versions of the PG-SGA, as this gives a unique opportunity to compare the obtained results between the studies. Secondly, the forward translations were performed by three persons instead of two, as in other studies [20, 22, 23], and the translated text was verified by 20 independent experts. Thanks to such a meticulous approach to the task, 32 differences in the translation of wordings were identified, which, after harmonization, allowed us to obtain the optimal version of the translation. Thirdly, we included a very large group of patients (*n* = 174) and HCPs (*n* = 188) in the cognitive debriefing and linguistic and content validation. To the best of our knowledge, this is the largest number of participants included in the PG-SGA linguistic and content validation studies to date. Fourthly, involvement of patients and HCPs from many centers located throughout Poland allowed us to consider our study as representative for the whole country. Finally, with a large group of HCPs and patients with different disease entities from different health care centers, differentiated in terms of gender, age, and education, our study sample is representative for the heterogeneous patient populations being at risk for malnutrition, and allowed for determining content validity, comprehensibility, and difficulty per type of HCP.

The study also has some limitations. The uneven distribution of numbers in the subgroups, both among patients and HCPs, could have influenced the results of the statistical analyses.

## Conclusions

Translation and cultural adaptation of the PG-SGA for the Polish setting according to ISPOR Principles preserved the purpose and conceptual meaning of the original PG-SGA. The linguistic and content validation revealed that the Polish version of PG-SGA is well understood and easy to complete by patients and professionals and considered relevant by professionals. However, detailed results for content validity, comprehensibility, and difficulty of the professional component of PG-SGA indicate the need for appropriate training of the Polish HCPs, especially physicians and nurses, mainly in the worksheets related to the metabolic demand and physical exam.

## Data Availability

No datasets were generated or analysed during the current study.
