# Correction to: Polish translation, cultural adaptation, and validity confirmation of the Scored Patient-Generated Subjective Global Assessment

**DOI:** 10.1007/s00520-024-08886-5

**Published:** 2024-09-27

**Authors:** Katarzyna Zabłocka-Słowińska, Joanna Pieczyńska, Anna Prescha, Maciej Bladowski, Damian Gajecki, Dorota Kamińska, Katarzyna Neubauer, Faith Ottery, Harriët Jager-Wittenaar

**Affiliations:** 1 The Faculty of Finance and Management, WSB Merito University in Wroclaw, Wroclaw, Poland; 2 Department of Health Sciences, University of Opole, Opole, Poland; 3 Department of Dietetics and Bromatology, Wroclaw Medical University, Wroclaw, Poland; 4 Department of Internal and Occupational Diseases, Hypertension and Clinical Oncology, Wroclaw Medical University, Wroclaw, Poland; 5 Department and Clinic of Nephrology and Transplantation Medicine, Wroclaw Medical University, Wroclaw, Poland; 6 Department and Clinic of Gastroenterology and Hepatology, Wroclaw Medical University, Wroclaw, Poland; 7 Ottery & Associates, LLC, Oncology Care Consultants, Chicago, USA; 8 Research Group Healthy Ageing, Allied Health Care and Nursing, Hanze University of Applied Sciences, Groningen, Netherlands; 9 Department of Gastroenterology and Hepatology, Dietetics, Radboud University Medical Center, Nijmegen, Netherlands; 10 Faculty of Physical Education and Physiotherapy, Department Physiotherapy and Human Anatomy, Research Unit Experimental Anatomy, Vrije Universiteit Brussel, Brussels, Belgium

**Correction to: Supportive Care in Cancer (2024)**

10.1007/s00520-024-08808-5

The correct affiliation of Joanna Pieczyńska and Anna Prescha is affiliation 3 which is:

Department of Dietetics and Bromatology, Wroclaw Medical University, Wroclaw, Poland

and not affiliation 1 which is:

The Faculty of Finance and Management, WSB Merito University in Wroclaw, Wroclaw, Poland

Orcid numbers are also added for both authors.

The original article has been corrected.

